# Metabolic and Epigenetic Reprogramming in a Case of Nuclear Protein in Testis (NUT) Carcinoma of the Retroperitoneum

**DOI:** 10.7759/cureus.52814

**Published:** 2024-01-23

**Authors:** Mika Serizawa, Kaho Serizawa, Kenta Masui, Makoto Toguchi, Kumiko Murakami, Tomoko Yamamoto, Yoji Nagashima, Toshio Takagi, Atsushi Kurata

**Affiliations:** 1 Department of Pathology, Tokyo Women's Medical University, Tokyo, JPN; 2 Department of Urology, Tokyo Women's Medical University Hospital, Tokyo, JPN; 3 Department of Surgical Pathology, Tokyo Women's Medical University Hospital, Tokyo, JPN

**Keywords:** retroperitoneum, epigenetics, metabolic reprogramming, brd4-nut, nut carcinoma

## Abstract

Nuclear protein in testis (NUT) carcinoma is a rare but highly aggressive carcinoma, driven by genetic rearrangement of the NUT midline carcinoma family member 1 (*NUTM1*) gene on chromosome 15q14. Recently, a tight link has been suggested between genetic abnormalities and subsequent metabolic and epigenetic dysregulation to drive the progression of malignant tumors. However, it remains elusive whether such reprogramming could contribute to the pathogenesis of NUT carcinoma. We herein report an autopsy case of NUT carcinoma arising in the retroperitoneum of a 31-year-old male. Notably, reprogramming of glycolytic metabolism and epigenetic histone modifications was observed in this unusual NUT carcinoma case, and this phenomenon was further confirmed by an in vitro cell culture model with bromodomain containing 4 (*BRD4*)-*NUT* overexpression. The rationale for documenting the case is based on our findings to reveal that metabolic and epigenetic reprogramming could be one of the contributing factors to the pathogenesis of NUT carcinoma, which could be exploitable as a novel therapeutic target for this rare and aggressive cancer type.

## Introduction

Nuclear protein in testis (NUT) carcinoma is a poorly differentiated type of carcinoma, driven by genetic rearrangement of the *NUT* gene on chromosome (chr) 15q14. Originally, this cancer was named t(15;19) carcinoma or NUT midline carcinoma according to its representative genetic aberration as well as the anatomic location of midline structures such as the head, neck, and thorax including the thymus [1]. Subsequently, the tumor has been reported to arise even outside the midline, and more importantly, around 70% of cases were found to possess a pathognomonic bromodomain containing 4 *(BRD4)-NUT* fusion gene, a reciprocal translocation between the NUT midline carcinoma family member 1 (*NUTM1*) gene on chr 15q14 and *BRD4* on chr 19p13.1 [2]. Still, how the *BRD4-NUT* fusion underlies the pathogenesis of NUT carcinoma remains elusive.

Metabolic reprogramming is an emerging, core hallmark of cancer that meets an energetic demand for proliferating tumor cells [3]. Recent studies demonstrated that metabolic shifts in cancer could be involved with neoplastic development via epigenetic changes, including histone modifications [4]. Of interest, in NUT carcinoma, *BRD4-NUT* forms p300 histone acetyltransferase-dependent nuclear foci characterized by an accumulation of acetylated histones [5], which could lead to a genome-wide shift in the epigenetic landscape of the tumor. Thus, genetic hits could initiate the formation of NUT carcinoma, and subsequent epigenetic dysregulation may drive its progression via metabolic reprogramming.

We herein report a rare autopsy case of NUT carcinoma in the retroperitoneum, identifying specific metabolic and epigenetic reprogramming in this primary human tumor further examined through a cell culture model. The findings indicate that a dynamic shift of glycolysis and histone acetylation could be the basis for the aggressiveness of NUT carcinoma as well as its promising druggable target.

## Case presentation

**Figure 1:**
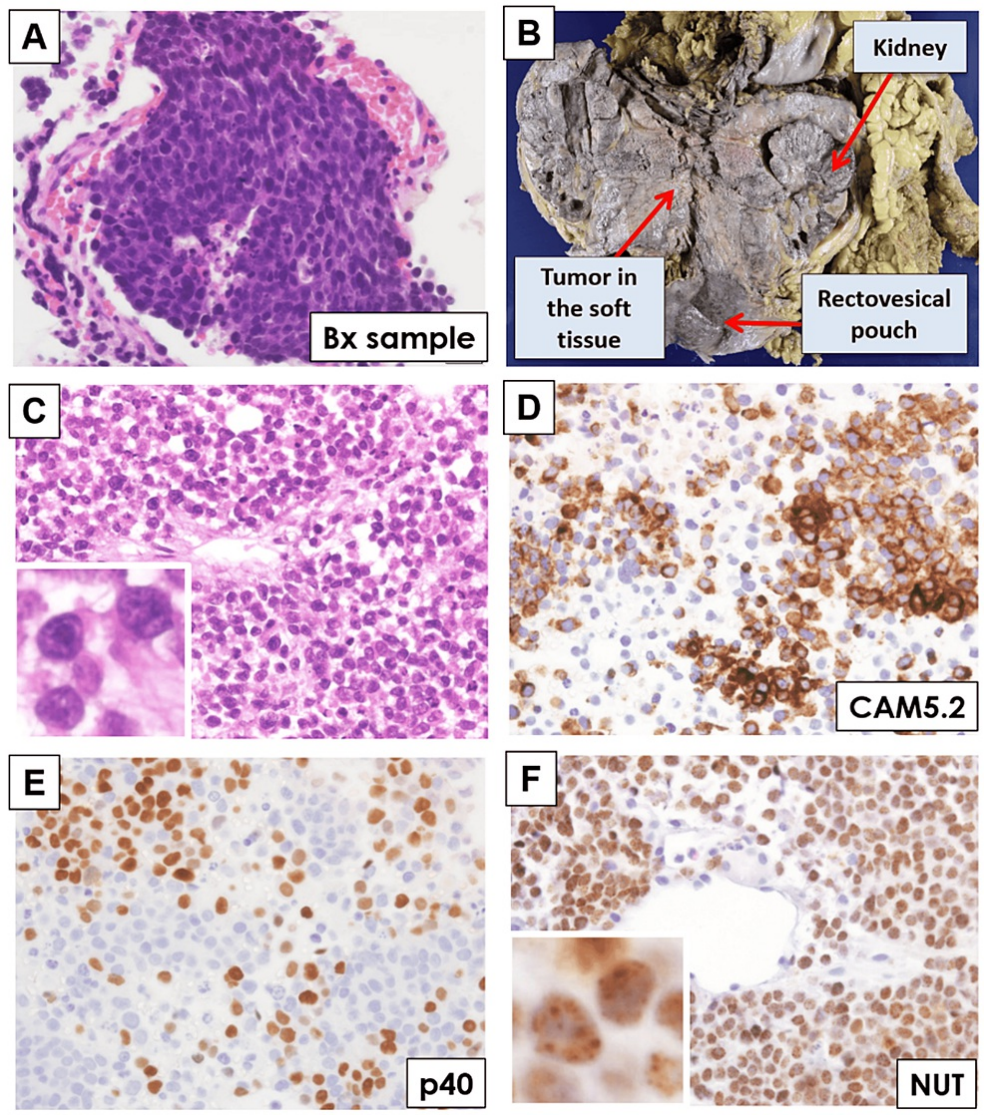
Biopsy and autopsy samples of human NUT carcinoma in the retroperitoneum A: CT-guided biopsy sample of the tumor showed dense proliferation of small atypical cells with a high nucleocytoplasmic ratio. Bx, biopsy. B: A macroscopic view of the autopsied sample demonstrated a very large tumor (24 x 15 x 10 cm) in the retroperitoneum which involved the left kidney and extended to the rectovesical pouch. C: The tumor from the autopsy sample showed a dense proliferation of monomorphic, round tumor cells with a high nucleocytoplasmic ratio. Inset, magnified view of tumor nuclei with fine chromatin and prominent nucleoli. D, E: Tumor cells are immunopositive for cytokeratin (clone CAM5.2) (D) and p40 (E), indicating its histology as poorly differentiated squamous cell carcinoma. F: Tumor cells demonstrated diffuse nuclear reactivity for *NUT* protein. Inset, magnified view of NUT positivity with a speckled pattern.

A 31-year-old male patient had a 24-year history of systemic lupus erythematosus (SLE), and his renal failure due to lupus nephritis necessitated hemodialysis and subsequent right renal transplantation from a living donor. The patient is currently maintained on an immunosuppression regimen that consists of methylprednisolone (16 mg/day) and tacrolimus (1 mg/day). The patient has no specific family history that would indicate a predisposition to either SLE or germline cancer syndrome. Along the course of his regular follow-up, chest/abdomen/pelvis uncontrasted CT detected an ill-defined 10+ cm soft tissue density with intratumoral hemorrhage in the upper portion of the left kidney. Biochemical examination in the blood revealed that tumor markers were slightly elevated, including carbohydrate antigen 125 (82 U/mL: normal <35) and neuron-specific enolase (92.8 ng/mL: normal <16.3). A CT-guided-guided was performed, and a tiny fragment of tumor tissue displayed dense proliferation of small atypical cells with a high nucleocytoplasmic ratio, giving the findings of a so-called small blue round cell tumor (Figure 1A). Its definitive diagnosis was difficult due to a lack of sufficient tumor tissue within the sample, and a tentative pathological diagnosis of poorly differentiated carcinoma most likely representing a small round blue cell tumor with a fairly wide differential diagnosis was rendered, considering its morphology and an immunophenotype that included a positivity for pancytokeratin (AE1/AE3), epithelial membrane antigen, CD99, and synaptophysin, and a high Ki-67/MIB-1 proliferation index (70-80%). Then, the tumor progressed rapidly, and pazopanib (a multi-target inhibitor including vascular endothelial growth factor: 400 mg/day) was administered but did not affect the tumor. Hospital care was provided to the patient, and he died two months after his initial diagnosis of the malignancy.

Subsequently, an autopsy was performed to determine the final diagnosis of the tumor. A very large tumor lesion (24 x 15 x 10 cm) was found in the retroperitoneum, which involved the left kidney and extended to the rectovesical pouch (Figure 1B). The tumor invaded the adjacent structures, i.e., the jejunum, rectum, urinary bladder, aorta, and inferior vena cava, and metastasized to periaortic lymph nodes. Histologically, the tumor showed a sheet-like proliferation of monomorphic, round-to-polygonal tumor cells with a high nucleocytoplasmic ratio, providing an impression of undifferentiated malignant tumors (Figure 1C). Immunostained tumor cells expressed cytokeratin (clones AE1/AE3, CAM5.2) and a squamous cell marker, p40, but were negative for spalt like transcription factor 4, desmin, Wilms tumor 1, melan A, CD3, or CD20, suggesting its histology as poorly differentiated carcinoma with squamous differentiation (Figure 1D-1E). Notably, tumor cells demonstrated diffuse nuclear reactivity for *NUT* protein (C52B1: Cell Signaling Technology, Danvers, MA) with a pathognomonic, speckled pattern (Figure 1F) [6]. Thus, the final pathology diagnosis was a NUT carcinoma, with a presumed primary site of origin as the retroperitoneum.

**Figure 2:**
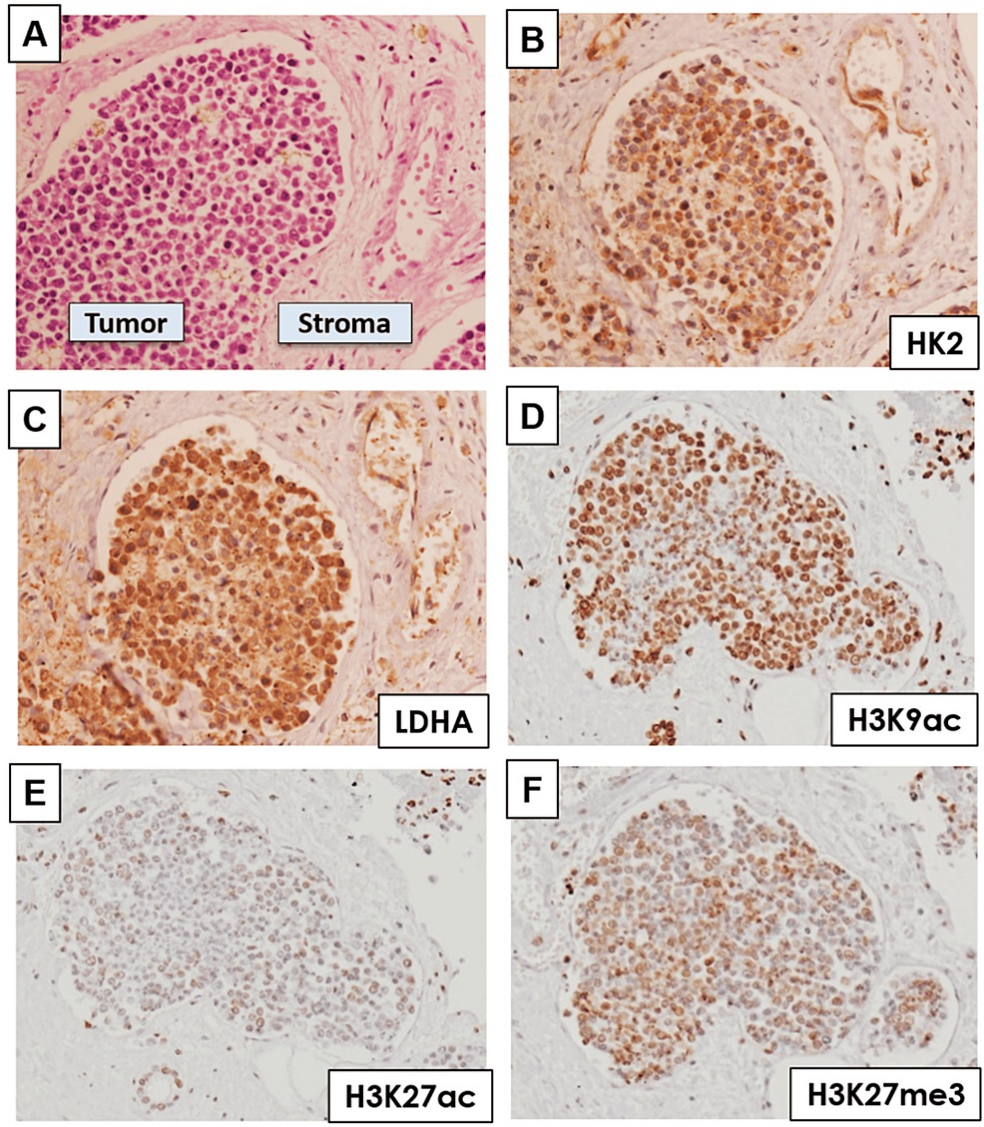
Metabolic and epigenetic reprogramming in human NUT carcinoma case A: Section shows both components of NUT carcinoma tissue and surrounding stromal tissue. B, C: NUT carcinoma cells are strongly immunopositive for glycolytic markers of HK2 (B) and LDHA (C). D-F: In accordance with metabolic shift in human NUT carcinoma tissue, oncogenic histone acetylation marks of H3K9ac (D) and H3K27ac (E) as well as histone methylation marks of H3K27me3 (F) were upregulated in NUT carcinoma. H3K9ac was most prominently expressed in the tumor tissue (D).

Given the cellular function of the *NUT* gene fusion partner *BRD4* being metabolic and epigenetic regulation, we went on to examine how this gene fusion influenced metabolic reprogramming within the NUT carcinoma. We first assessed the expression of glycolytic enzymes in a human autopsied case of NUT carcinoma by immunohistochemistry. Of note, glycolytic enzymes such as hexokinase 2 (HK2) and lactate dehydrogenase (LDHA) were overexpressed in NUT carcinoma tissue compared with surrounding stroma, suggesting that glycolytic metabolism was activated in NUT carcinoma (Figure 2A-2C). A series of studies reported that metabolic reprogramming modifies cancer biology by affecting histone modifications such as acetylation and methylation [4,7]. In consideration of a metabolic shift in human NUT carcinoma tissue, we next assessed its histone modification status, including methylation and acetylation. We found that both histone markers of tumor-promoting acetylation and methylation were upregulated in NUT carcinoma tissue in comparison with those in the surrounding stroma, indicating a tumor-specific epigenetic shift in NUT carcinoma (Figure 2D-2F) [8,9]. Of note, H3K9 acetylation (H3K9ac) was most prominently expressed in the tumor tissue (Figure 2D).

Together, these suggest that metabolic and epigenetic reprogramming can be actively induced in human NUT carcinoma, as was documented in this case. We then examined whether a shift in metabolism and histone modifications is directly associated with the expression of oncogenes in NUT carcinoma. To test the hypothesis, we exploited the cell culture system with an artificial overexpression of the pathognomonic fusion for NUT carcinoma, *BRD4-NUT*. Detailed information on the methods of cell culture analysis is described in Table 1.

**Table 1:** Methodological investigation in cell culture analysis

Methods for cell culture analysis	
Cell culture	293T cells were cultured in DMEM (Thermo Fisher; Waltham, MA) supplemented with 10% FBS (Omega Scientific; Tarzana, CA) and 1% penicillin-streptomycin (PS) (Thermo Fisher).
DNA plasmid transfection	Transfections of *GFP* and *BRD4-NUT* DNA plasmids were performed using X-tremeGENE HP (Roche; Basle, Switzerland), and cells were typically harvested 48 hours post-transfection.
Immunostaining	Immunostaining was performed as previously described [8,9]. Slides were counterstained with hematoxylin or DAPI (Thermo Fisher) to visualize nuclei. Immunostained sections underwent immunohistochemical analysis in which the results were evaluated independently by two pathologists who were unaware of the findings of the molecular analyses. Immunofluorescent samples were analyzed with a fluorescent microscope (Olympus BX53 Digital Fluorescence Microscope).
Western blot	Immunoblotting was performed as described previously [8,9]. Cells were lysed and homogenized with radioimmunoprecipitation assay (RIPA) lysis buffer from Boston BioProducts (Boston, MA). Equal amounts of protein extracts (30 μg) were separated by electrophoresis on 4-20% Mini-PROTEAN TGX Precast Gels (Bio-Rad; Hercules, CA) and then transferred to a nitrocellulose membrane with Trans-Blot Turbo Transfer System (Bio-Rad). The membrane was probed with the primary antibodies (1:1000), followed by HRP-conjugated secondary antibodies (1:5000) (Cell Signaling Technology; Danvers, MA). The immunoreactivity was detected with Super Signal West Pico Chemiluminescent Substrate in combination with the West Femto Trial kit (Thermo Fisher). Quantitative densitometry analysis was performed with image analysis software (ImageJ version 1.49, NIH).
Cell proliferation assay	*GFP* or *BRD4-NUT* overexpressing 293T cells were seeded (4.0×105 cells per well of 6-well dish) in a glucose-containing medium (DMEM) with 10% FBS and 1% PS for 24 hours (n=6). Cell proliferation was measured by cell counting with an automated cell counter (TC20TM; Bio-Rad).
Statistical analysis	Statistical differences between the two groups were analyzed using Student's two-tailed unpaired t-test. Error bars represented standard deviation (SD), and statistical significance was indicated as *p<0.05, **p<0.01, and ***p<0.001.

**Figure 3:**
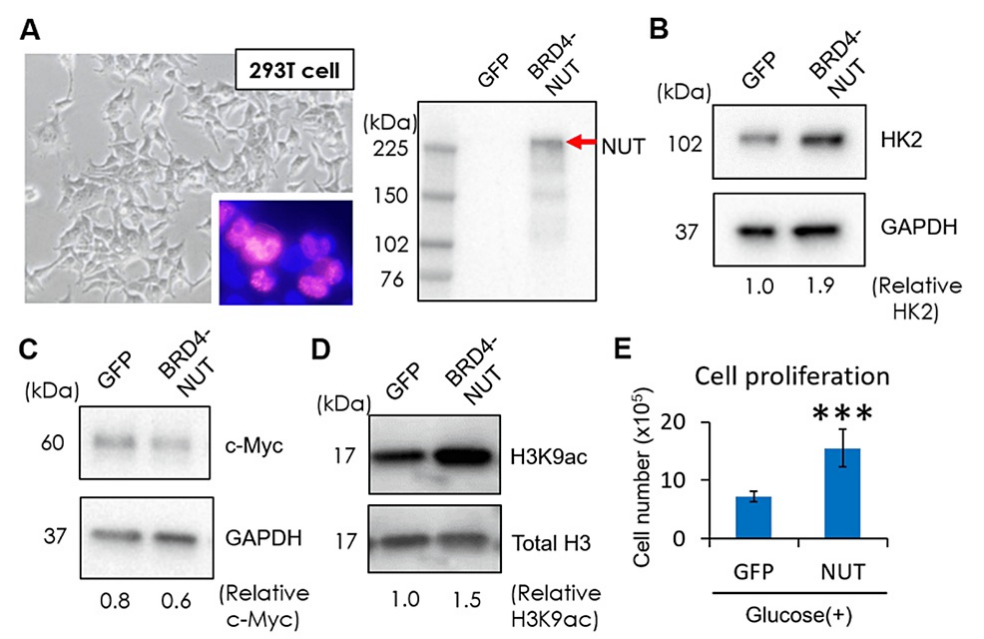
Metabolic and epigenetic reprogramming in NUT-overexpressing cell culture model A: 293T cells overexpressed with *BRD4-NUT* plasmid. Successful overexpression of *BRD4-NUT* was confirmed by staining NUT with immunocytochemistry (red, NUT; blue, 4′,6-diamidino-2-phenylindole (DAPI)) and immunoblotting. GFP, *GFP*-overexpressing control 293T cells; BRD4-NUT, *BRD4-NUT*-overexpressing 293T cells. B: Immunoblot detection of glycolytic enzymes (HK2) in *BRD4-NUT* overexpressing cells versus control cells with *GFP* plasmid. Glyceraldehyde-3-phosphate dehydrogenase (GAPDH), loading control. C: Immunoblot detection of c-Myc (MYC) in *BRD4-NUT* overexpressing cells versus control cells with *GFP* plasmid. GAPDH, loading control. D: Immunoblot detection of histone H3 acetylation (H3K9ac) in *BRD4-NUT* overexpressing cells versus control cells with *GFP* plasmid. Total H3, loading control. E: Cells were seeded at the concentration of 4.0 x 10^5^ cells/well, and cell number was calculated 24 hours after transfection between *BRD4-NUT* overexpressing cells vs. control cells with *GFP* plasmid in glucose-containing media (n=6 (six separate transfection experiments on the same day by two people)). ***p<0.001.

We successfully overexpressed the plasmid of *BRD4-NUT* (pcDNA5 frt/to N-BioTAP-C-BRD4-NUT: Addgene, Watertown, MA) in HEK293T embryonic kidney cells (ATCC, Manassas, VA) (Figure 3A) and found that glycolytic enzymes were upregulated in *BRD4-NUT* overexpressing cells in contrast to control cells with green fluorescent protein (*GFP*) plasmid, suggesting that *BRD4-NUT* fusion protein could activate glycolytic metabolism (Figure 3B). We previously demonstrated that c-Myc (MYC) is a master regulator of glycolytic metabolism in malignant tumors [10], but the overexpression of *BRD4-NUT* did not have an impact on the induction of MYC in our model, at least at the protein level (Figure 3C). Similar to human NUT carcinoma tissue, the overexpression of *BRD4-NUT* significantly upregulated histone methylation and acetylation, especially H3K9ac, indicating that a tumor-specific epigenetic shift could be induced by the *BRD4-NUT* fusion protein (Figure 3D). More importantly, overexpression of *NUT* could significantly drive cell proliferation in glucose-containing media (Figure 3E).

**Figure 4:**
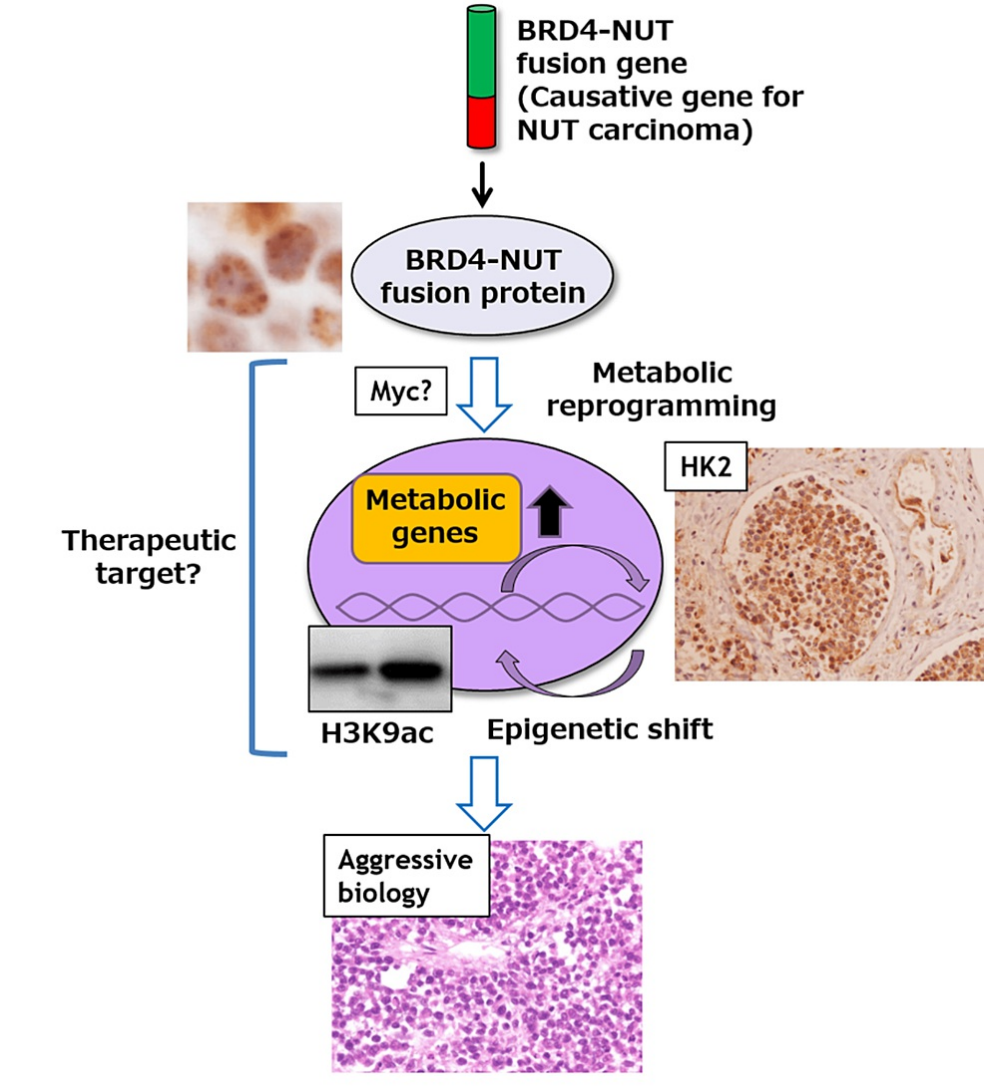
BRD4-NUT drives metabolic and epigenetic reprogramming in NUT carcinoma *BRD4-NUT* protein, produced by *BRD4-NUT* fusion genes, could upregulate metabolic genes such as *HK2* and *LDHA *and reprogram glycolytic metabolism, which subsequently shifts tumor epigenome represented by an increase of H3K9ac. Metabolic and epigenetic reprogramming contributes to the aggressive biology of NUT carcinoma but could be exploitable as a novel therapeutic target for the rare and aggressive types of cancer.

Together, *BRD4-NUT* could regulate metabolic and epigenetic reprogramming for glucose-dependent cell survival in NUT carcinoma (Figure 4).

## Discussion

NUT carcinoma is a rare malignant tumor, with its morphology ranging from keratinizing squamous cell carcinoma to undifferentiated tumors [1]. Further, although initially thought to arise at anatomic sites near the midline of the body (particularly the head and neck), it has now been identified outside the midline structures, including the salivary gland, lung, pancreas, kidney, soft tissue, and bone [1,11]. Significant variations in histology, as well as location, should make the definitive diagnosis of NUT carcinoma particularly difficult, especially if it occurs in atypical regions like ours. Indeed, the present case of NUT carcinoma demonstrated an unusual anatomical site of soft tissue around the left kidney, in combination with its undifferentiated morphology of a small blue round cell tumor with CD99 and synaptophysin positivity, which rendered its diagnosis of malignant renal tumors, most probable as undifferentiated carcinoma, including neuroendocrine/small cell carcinoma. Of interest, NUT carcinoma frequently expresses squamous cell carcinoma markers (p40 and cytokeratin 5/6) even if its morphology is undifferentiated [12], and immunohistochemistry for *NUT* protein with a nuclear-speckled pattern is also useful to establish a definitive diagnosis [6]. Thus, NUT carcinoma should be included as one of the differential diagnoses for undifferentiated or small cell carcinomas even in the “non-midline” location, combined with its molecular profiling with immunohistochemistry or fluorescence in situ hybridization [13], with a wide differential diagnosis that includes small cell carcinoma, Merkel cell carcinoma, and Epstein-Barr virus-related undifferentiated carcinoma, among others, which could particularly render the diagnosis of NUT carcinoma complicated and challenging due to its undifferentiated nature [1,12].

Contributing factors to tumorigenesis and malignant transformation in NUT carcinoma should be based upon the pathognomonic genetic aberration of *BRD4-NUT* fusions or other rare fusion variants, including *NUTM1* fused to a gene of bromodomain containing 3 (*BRD3*), nuclear receptor binding SET domain protein 3 (*NSD3*), and others [1]. However, it has remained unclear how *NUT* fusion genes could contribute to the aggressive biology of NUT carcinoma. Recent studies demonstrated a tight link between genetic abnormality and subsequent metabolic/epigenetic dysregulation, which could drive the progression of malignant tumors [4]. In NUT carcinoma, *BRD4-NUT* was reported to co-localize with active histone marks (H3K9ac, H3K14ac, and H3K27ac) in a p300-dependent manner, epigenetically regulating the expression of the oncogenic transcription factor MYC [5,14]. Of note, a dynamic epigenetic shift is driven by aerobic glycolysis known as the Warburg effect or metabolic reprogramming [4,7], and we thus hypothesized that metabolic and epigenetic reprogramming could facilitate the aggressive biology of NUT carcinoma. Our results corroborate the idea that metabolic reprogramming and subsequent epigenetic changes could contribute to glucose-dependent cell survival of NUT carcinoma in a *BRD4-NUT* gene fusion product-dependent manner. The limitation of the study is that our cell culture system exploited non-NUT carcinoma cells, which might be negatively affected by *BRD4-NUT* overexpression [1]. Future endeavors are thus necessary to examine the metabolic contribution to NUT carcinoma biology with a human-derived NUT carcinoma primary cell culture model, which, to the best of our knowledge, has yet to be established, in combination with its secondary cell line panel [15].

No effective treatment for NUT carcinoma has been reported so far, and the patient being presented in this case report succumbed to the disease two months after his diagnosis. Novel therapeutic strategies are now under investigation, and the epigenetic shift could be one of the druggable targets against NUT carcinoma. Bromodomain and extraterminal domain (BET) inhibitors such as JQ1 directly inhibit the action of BRD4 by competitively inhibiting the binding of BET bromodomains to histone-acetylated lysines [1,16]. Furthermore, emerging therapeutic modalities of proteolysis targeting chimera (PROTACs) could be applied to the treatment of NUT carcinoma by BET-targeting PROTACs such as cereblon-dependent BET protein degradation (dBETs) and *NUTM1*-interference with nanobodies or small molecular ligands [17]. Histone deacetylase inhibitors (HDACi) are potentially another type of epigenetic modifier for targeting NUT carcinoma, inducing squamous differentiation of NUT carcinoma cells [18]. Indeed, some classes of HDACi have been examined clinically, especially in combination with cytotoxic chemotherapy or other molecular targeting drugs [19,20]. These studies suggest that epigenetics in NUT carcinoma could be therapeutically, targetable as we also demonstrated the same trend in other types of malignant tumors (Figure 4) [8,9]. Additionally, metabolism itself could also be a promising druggable target, and combinatorial approaches of conventional cytotoxic drugs and modifiers of metabolism should be the next to be examined for novel therapeutic strategies against NUT carcinoma (Figure 4) [21]. This should be screened with histology-oriented approaches to “metabolism-based pathology” [22], as well as the development of specific in vitro or in vivo models to precisely recapitulate the aggressive biology of human NUT carcinoma.

## Conclusions

We report an autopsy case of NUT carcinoma arising in the retroperitoneum of a 31-year-old male SLE patient. Reprogramming of glycolytic metabolism and tumor epigenome was observed in this unusual NUT carcinoma case, as well as an in vitro *BRD4-NUT* overexpression system, which could be exploitable as a novel therapeutic target for these rare and aggressive types of cancer.

Although novel therapeutic strategies against NUT carcinoma are now under investigation, we demonstrated that metabolism and epigenetics could be a promising druggable target as well as its diagnostic marker with histology-oriented approaches to metabolism-based pathology. Our case report thus indicates the importance of clinically recognizing and investigating the metabolic activities of NUT carcinoma to provide better care to patients and improve their overall survival.

## References

[1] French CA (2018). NUT carcinoma: clinicopathologic features, pathogenesis, and treatment. Pathol Int.

[2] French CA, Miyoshi I, Kubonishi I, Grier HE, Perez-Atayde AR, Fletcher JA (2003). BRD4-NUT fusion oncogene: a novel mechanism in aggressive carcinoma. Cancer Res.

[3] Pavlova NN, Thompson CB (2016). The emerging hallmarks of cancer metabolism. Cell Metab.

[4] Masui K, Harachi M, K Cavenee W, S Mischel P, Shibata N (2020). Codependency of metabolism and epigenetics drives cancer progression: a review. Acta Histochem Cytochem.

[5] Reynoird N, Schwartz BE, Delvecchio M (2010). Oncogenesis by sequestration of CBP/p300 in transcriptionally inactive hyperacetylated chromatin domains. EMBO J.

[6] Haack H, Johnson LA, Fry CJ (2009). Diagnosis of NUT midline carcinoma using a NUT-specific monoclonal antibody. Am J Surg Pathol.

[7] Kaelin WG Jr, McKnight SL (2013). Influence of metabolism on epigenetics and disease. Cell.

[8] Harachi M, Masui K, Honda H (2020). Dual regulation of histone methylation by mTOR complexes controls glioblastoma tumor cell growth via EZH2 and Sam. Mol Cancer Res.

[9] Masui K, Harachi M, Ikegami S (2019). mTORC2 links growth factor signaling with epigenetic regulation of iron metabolism in glioblastoma. J Biol Chem.

[10] Masui K, Tanaka K, Akhavan D (2013). mTOR complex 2 controls glycolytic metabolism in glioblastoma through FoxO acetylation and upregulation of c-Myc. Cell Metab.

[11] Bauer DE, Mitchell CM, Strait KM (2012). Clinicopathologic features and long-term outcomes of NUT midline carcinoma. Clin Cancer Res.

[12] Tilson MP, Bishop JA (2014). Utility of p40 in the differential diagnosis of small round blue cell tumors of the sinonasal tract. Head Neck Pathol.

[13] French CA, Kutok JL, Faquin WC (2004). Midline carcinoma of children and young adults with NUT rearrangement. J Clin Oncol.

[14] Grayson AR, Walsh EM, Cameron MJ (2014). MYC, a downstream target of BRD-NUT, is necessary and sufficient for the blockade of differentiation in NUT midline carcinoma. Oncogene.

[15] Stirnweiss A, Oommen J, Kotecha RS, Kees UR, Beesley AH (2017). Molecular-genetic profiling and high-throughput in vitro drug screening in NUT midline carcinoma-an aggressive and fatal disease. Oncotarget.

[16] Filippakopoulos P, Qi J, Picaud S (2010). Selective inhibition of BET bromodomains. Nature.

[17] Hakun MC, Gu B (2021). Challenges and opportunities in NUT carcinoma research. Genes (Basel).

[18] Schwartz BE, Hofer MD, Lemieux ME (2011). Differentiation of NUT midline carcinoma by epigenomic reprogramming. Cancer Res.

[19] Maher OM, Christensen AM, Yedururi S, Bell D, Tarek N (2015). Histone deacetylase inhibitor for NUT midline carcinoma. Pediatr Blood Cancer.

[20] Beesley AH, Stirnweiss A, Ferrari E (2014). Comparative drug screening in NUT midline carcinoma. Br J Cancer.

[21] French CA, Cheng ML, Hanna GJ (2022). Report of the first international symposium on NUT carcinoma. Clin Cancer Res.

[22] Masui K, Mischel PS (2023). Metabolic and epigenetic reprogramming in the pathogenesis of glioblastoma: toward the establishment of "metabolism-based pathology". Pathol Int.

